# *Echium amoenum* and Rosmarinic Acid Suppress the Growth and Metastasis of Gastric Cancer AGS Cells by Promoting Apoptosis and Inhibiting EMT

**DOI:** 10.3390/ijms252312909

**Published:** 2024-11-30

**Authors:** Mahdieh Ahmadi, Hong Lae Kim, So Jin Park, Hye Jin Jung

**Affiliations:** 1 Department of Life Science and Biochemical Engineering, Graduate School, Sun Moon University, Asan 31460, Republic of Korea; mahtiti77@naver.com (M.A.); llee5405@gmail.com (H.L.K.); psj1867@naver.com (S.J.P.); 2 Department of Pharmaceutical Engineering and Biotechnology, Sun Moon University, Asan 31460, Republic of Korea

**Keywords:** gastric cancer, *Echium amoenum*, rosmarinic acid, apoptosis, epithelial–mesenchymal transition

## Abstract

Gastric cancer (GC) ranks as the fifth most prevalent cancer globally. Owing to the absence of early manifest symptoms, it is difficult to diagnose GC until it has metastasized to other organs. Hence, the prevention and treatment of GC have become major concerns for patients. *Echium amoenum*, a traditional medicinal plant from the Boraginaceae family, exhibits various biological activities. Although recent studies have reported the anticancer properties of *E. amoenum*, its effects and mechanisms of action on GC cells are not yet fully understood. This study examined the anticancer effects of the ethyl acetate extract of *E. amoenum* (EAEC) and its main active ingredient, rosmarinic acid (RA), in GC AGS cells. EAEC and RA suppressed AGS cell growth by inducing apoptosis through caspase mediation and inhibited AGS cell metastasis by influencing the expression of crucial epithelial–mesenchymal transition (EMT) biomarkers. Furthermore, the anti-growth and anti-metastatic effects of EAEC and RA on AGS cells involved inactivation of the STAT3, AKT, and ERK1/2 pathways. Additionally, RA notably inhibited the in vivo tumor growth in AGS cells. Overall, these results indicate that EAEC and RA could serve as potential anticancer and anti-metastasis agents for GC.

## 1. Introduction

Gastric cancer (GC) is the fifth most prevalent cancer and the third most common type of malignancy, making it a major cause of cancer-related deaths globally [1]. It is triggered by several factors, both pathogenic, such as infection with *Helicobacter pylori*, and non-pathogenic, such as mutations of the E-cadherin gene (*CDH1*), genetic factors, interleukin gene polymorphisms, poor diet, and smoking [2,3]. Although various treatment methods, including surgery, chemotherapy, and endoscopy, exist for GC, the high toxicity, major side effects, and low efficacy of current drugs pose serious challenges for patients [3,4]. Therefore, the development of highly effective drugs is crucial for the care of patients with GC.

Natural products have long been used to prevent and treat various diseases. Numerous studies have reported that various natural products exhibit anticancer activities [5]. *Echium amoenum*, also known as borage, is a plant of the Boraginaceae family; it is mostly found in northern Iran, the Mediterranean, and different regions of Europe [6]. The dry petals of *E. amoenum* have various anti-inflammatory, sedative, mood-enhancing, stress-relieving, antidepressant, and anticancer properties [7]. In a previous study, the water extract of *E. amoenum* exerted cytotoxic effects on HepG2 liver cancer cells via damage to the mitochondria and lysosomes resulting from oxidative stress and reduction in glutathione levels [8]. In addition, the cytotoxic effect of the water extract of *E. amoenum* on human glioblastoma cells has been reported. [9]. However, no studies have yet investigated the effects of the ethyl acetate extract of *E. amoenum* (EAEC) on GC cells. *E. amoenum* contains several bioactive compounds, such as rosmarinic acid (RA), echimidine, echimidine isomer, 7-angeloylretronecine, 7-tigloylretronecine, anthocyanidins, flavonoids, alkaloids, saponins, unsaturated terpenoids, and sterols [10,11]. Another study reported that the major compound in *E. amoenum* is RA, a water-soluble phenolic compound that exists in herbal plants [12]. Multiple bioactivities of RA, including antioxidant, antimutagenic, and pro-apoptotic effects, have been reported [13,14]. RA also exhibits anti-angiogenic potential by lowering the intracellular levels of reactive oxygen species (ROS), H_2_O_2-_dependent vascular endothelial growth factor (VEGF) levels, and interleukin-8 (IL-8) release in endothelial cells [15]. RA has shown beneficial effects in the treatment of several cancers, including GC [16]. It inhibits proliferation and the Warburg effect in MKN45 GC cells by regulating the inflammation pathway and microRNA (miR)-155 [17]. In addition, RA inhibits SGC-7901 GC cells through the mitochondrial apoptosis pathway [18]. RA also increases the chemosensitivity of resistant SGC7901 GC cells to 5-fluorouracil by downregulating miR-6785-5p and miR-642a-3p and upregulating FOXO4 expression [19]. This evidence supports the antiproliferative effects of RA on GC cells. However, the anti-metastatic effects of RA and the molecular mechanisms underlying these effects in GC cells remain insufficiently explored. Thus, in this study, we examined the impact of EAEC and its major compound, RA, on the growth and metastasis of AGS GC cells.

Apoptosis is a tightly controlled programmed cell death process that occurs as a result of cellular damage [20]. It plays a significant role in immune system modulation and is closely linked to cancer [21]. Therefore, targeting cancer cells by inducing nuclear damage and apoptosis is a highly effective strategy in various cancer therapies, including chemotherapy. Apoptosis involves two pathways—intrinsic and extrinsic. The intrinsic pathway pertains to cellular stress, while the extrinsic pathway is activated by signals from neighboring cells. These pathways mediate the activation of caspases, leading to cell death [20,21]. Thus, herbal compounds and their active ingredients can enhance cancer treatment by activating the apoptotic pathways in GC cells.

Metastasis is the primary cause of mortality due to GC and is a complicated process that encompasses various mechanisms, such as the epithelial–mesenchymal transition (EMT) of GC cells, wherein cancer cells lose their polarity and intercellular adhesion, acquiring mesenchymal properties that lead to functional changes in cell migration and invasion [22]. Several signaling pathways initiate EMT, including Janus kinase/signal transducer and activator of transcription 3 (JAK/STAT3), phosphatidylinositol 3-kinase/protein kinase B (PI3K/AKT), and mitogen-activated protein kinase/extracellular signal-regulated kinase (MAPK/ERK). They induce the activation of EMT-specific transcription factors like Twist, Slug, Snail, and Zeb1, which in turn regulate the expression of EMT markers, including E-cadherin, N-cadherin, and vimentin [22,23,24]. Thus, targeting key regulators of EMT could be a promising strategy for preventing metastasis in GC. The results indicated that EAEC and RA have potential to serve as anticancer and anti-metastatic agents against GC by inducing apoptosis and blocking the EMT, migration, and invasion of GC cells.

## 2. Results

### 2.1. EAEC Prevents the Proliferation and Colony Formation of AGS Cells

**Figure 1:**
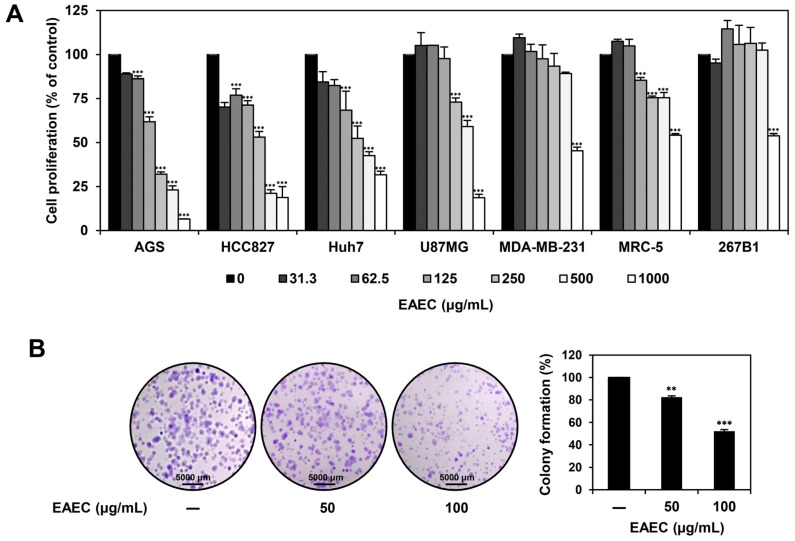
Effects of EAEC on the proliferation of various cancer and normal cell lines. (**A**) AGS, HCC827, Huh7, U87MG, MDA-MB-231, MRC-5, and 267B1 cells were exposed to increasing concentrations of EAEC (0–1000 μg/mL) for 72 h, and cell proliferation was assessed using the MTT assay. (**B**) AGS cells were exposed to EAEC (50, 100 μg/mL) and incubated for 6 days. Cell colonies were then stained with crystal violet. ** *p* < 0.01, *** *p* < 0.001 compared to control.

To determine the antiproliferative effect of EAEC, the MTT assay was performed on AGS (gastric cancer), HCC827 (lung cancer), Huh7 (liver cancer), U87MG (glioblastoma), MDA-MB-231 (breast cancer), MRC-5 (normal lung), and 267B1 (normal prostate) cell lines. The cells were exposed to different concentrations of EAEC (0–1000 μg/mL) for 72 h (Figure 1A). EAEC treatment reduced the proliferation of all cell lines, resulting in the IC_50_ values of 166.5, 327.5, 336.8, 852.4, 944.8, >1000, and >1000 μg/mL, respectively. It exerted the strongest inhibitory effect on AGS cell proliferation, with the lowest IC_50_ value. Next, the impact of EAEC treatment on AGS cell colony formation was assessed (Figure 1B). EAEC (50, 100 μg/mL) treatment suppressed AGS clonogenic proliferation in a concentration-dependent manner. These findings suggest that EAEC reduces the proliferation and colony-forming ability of AGS cells.

### 2.2. Analysis of the Active Ingredient of EAEC

**Figure 2:**
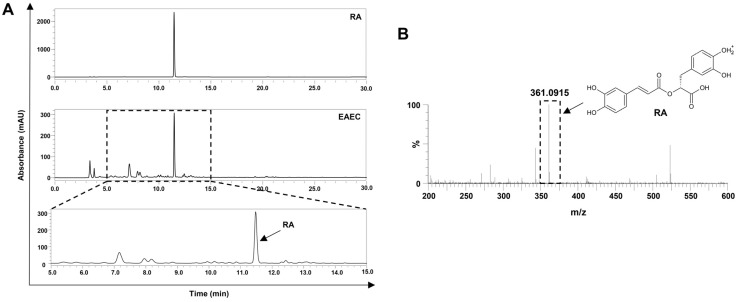
Analysis of the active ingredient in EAEC. (**A**) EAEC and the reference compound RA were analyzed using HPLC. (**B**) Identification of RA in EAEC via LC-MS analysis.

*E. amoenum* is a medicinal herb that contains several bioactive substances [25]. To identify the primary active ingredients of EAEC, high-performance liquid chromatography (HPLC) and liquid chromatography–mass spectrometry (LC-MS) analyses were performed. RA was identified at a retention time of 11.30 min, with a wavelength of 254 nm in the HPLC analysis of EAEC (Figure 2A). The concentration of RA, determined by HPLC, was 0.031 mg per 1 mg of EAEC. Moreover, RA was identified at a mass of 361.0915 m/z in the LC-MS analysis of EAEC (Figure 2B). These results confirm that RA is the principal active ingredient in EAEC.

### 2.3. RA Inhibits the Proliferation and Colony-Forming Ability of AGS Cells

**Figure 3:**
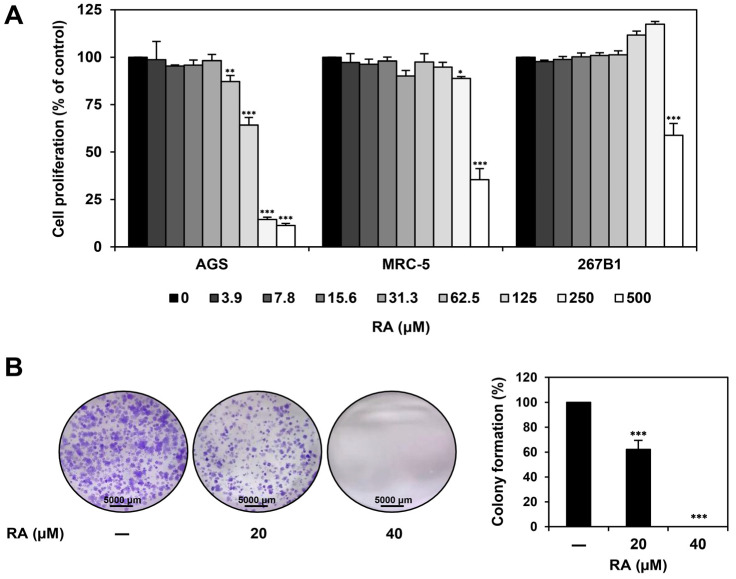
Effect of RA on the proliferation of AGS cells. (**A**) AGS, MRC-5, and 267B1 cells were exposed to increasing concentrations of RA (0–500 μM) for 72 h, and cell proliferation was assessed using the MTT assay. (**B**) AGS cells were exposed to RA (20, 40 μM) and incubated for 6 days. Cell colonies were then stained with crystal violet. * *p* < 0.05, ** *p* < 0.01, *** *p* < 0.001 compared to control.

To evaluate if the antiproliferative effect of EAEC on AGS cells is attributed to RA, the MTT assay was conducted. As depicted in Figure 3A, the cells were exposed to RA (0–500 μM) for 72 h. RA dose-dependently inhibited AGS cell proliferation, exhibiting an IC_50_ value of 140.4 μM. Specifically, RA showed a significantly stronger antiproliferative effect on AGS cells than on the normal cell lines MRC-5 and 267B1, with IC_50_ values of 426.5 μM and >500 μM, respectively. Furthermore, we demonstrated the inhibitory effect of RA on the colony-forming capacity of AGS cells. RA treatment (20, 40 μM) suppressed AGS colony formation in a concentration-dependent manner (Figure 3B). These data reveal that RA inhibits the proliferation and colony-forming ability of AGS cells.

### 2.4. EAEC and RA Trigger the Apoptotic Pathway Mediated by Caspases in AGS Cells

**Figure 4:**
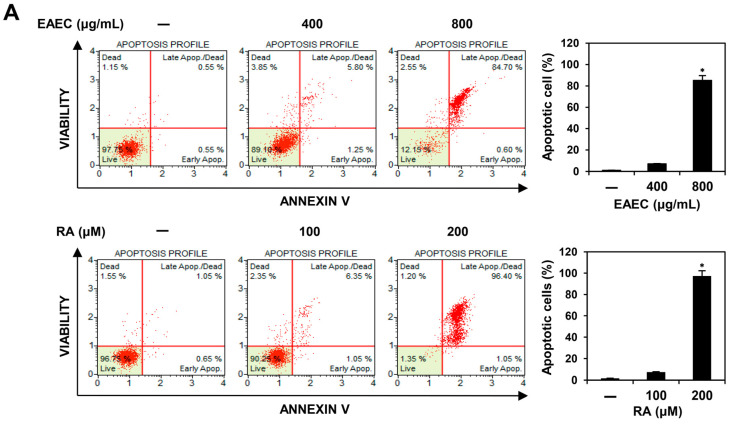

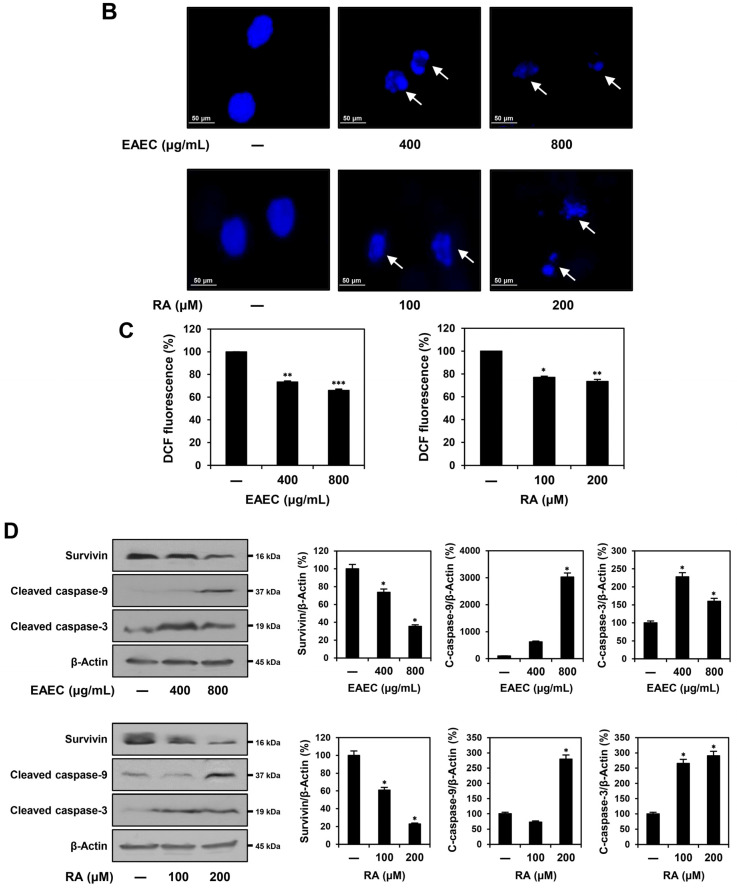
Effects of EAEC and RA on apoptotic cell death in AGS cells. (**A**–**D**) AGS cells were exposed to EAEC (400, 800 μg/mL) and RA (100, 200 μM) and incubated for 24 h. (**A**) The cells were stained using the Muse^®^ Annexin V & Dead Cell kit, and apoptotic cells were detected via flow cytometry. (**B**) AGS cell nuclei were stained with DAPI, and nuclear fragmentation (white arrows) was observed under a fluorescence microscope. (**C**) The effects of EAEC and RA on intracellular ROS generation were assessed by measuring ROS levels after staining with DCFH-DA using a microplate reader. (**D**) The expression levels of apoptosis regulators were evaluated through Western blot analysis. * *p* < 0.05, ** *p* < 0.01, *** *p* < 0.001 compared to control.

To investigate whether EAEC and RA inhibit cell proliferation by inducing apoptosis, AGS cells were labeled with Muse^®^ Annexin V & Dead Cell reagent and evaluated using flow cytometry. As shown in Figure 4A, compared with that in the AGS control group, treatment with EAEC (400, 800 μg/mL) increased early apoptosis to 0.60% of the total cells, while late apoptosis increased to 84.70% at higher concentration. Compared with the control group, the RA (100, 200 μM) treatment group showed an increase in early apoptosis to 1.05% and to 96.40% in late apoptosis at a higher concentration. To further investigate whether EAEC and RA have the potential to alter nuclear morphology, 4′,6-diamidine-2′-phenylindole dihydrochloride (DAPI) staining was performed. As illustrated in Figure 4B, treatment with EAEC (400, 800 μg/mL) and RA (100, 200 μM) notably induced nuclear fragmentation. Next, a ROS assay was performed using the fluorogenic indicator dichlorodihydrofluorescein diacetate (DCFH-DA). However, as shown in Figure 4C, treatment with EAEC and RA reduced ROS generation in AGS cells. Other studies have also reported the antioxidant activities of *E. amoenum* and RA [12,26]. Thus, the reduction in ROS levels in AGS cells may be related to their antioxidant effects. To further evaluate whether EAEC and RA regulate apoptosis in AGS cells, the levels of survivin and cleaved forms of caspase-9 and caspase-3 were examined. Survivin, an inhibitor of the apoptosis protein family, prevents apoptosis by inhibiting the activation of caspases [27]. Activated caspase-9 serves as the initiator protease in the intrinsic apoptotic pathway, cleaving and activating caspase-3 to execute cellular apoptosis [28]. Treatment with EAEC (400, 800 μg/mL) and RA (100, 200 μM) reduced the expression of survivin and increased that of cleaved caspase-9 and cleaved caspase-3 (Figure 4D). These findings indicate that the antiproliferative effects of EAEC and RA on AGS cells are linked to the induction of apoptosis.

### 2.5. EAEC and RA Suppress the Migratory and Invasive Abilities of AGS Cells

**Figure 5:**
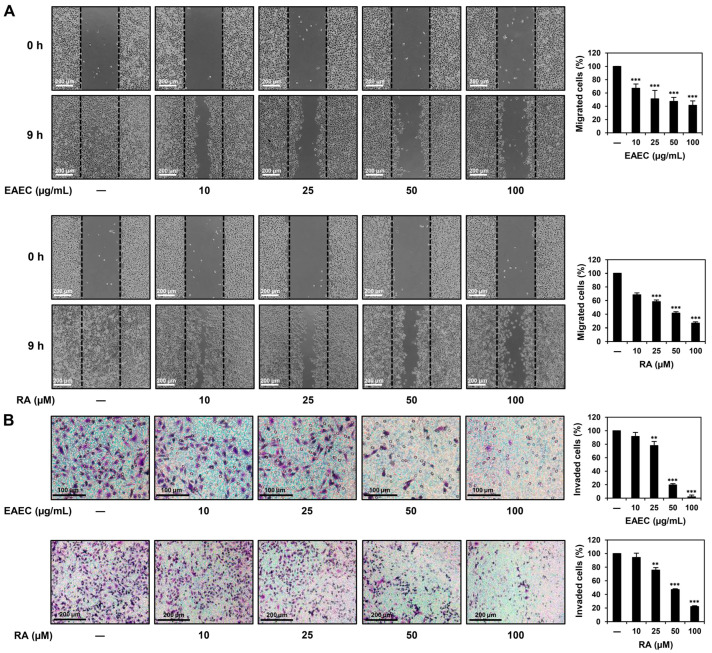
Effects of EAEC and RA on the migration and invasion abilities of AGS cells. (**A**) Migration was assessed using a wound closure assay. AGS cells were incubated for 9 h in the absence or presence of EAEC (10, 25, 50, 100 μg/mL) and RA (10, 25, 50, 100 μM). The migrated AGS cells within the gap were observed using an optical microscope. The black dashed lines represent the gap boundary at the initial time point. (**B**) Invasion was evaluated using a Transwell chamber with an ECM gel-coated membrane insert (8.0 μm pore size). AGS cells were exposed to EAEC (10, 25, 50, 100 μg/mL) and RA (10, 25, 50, 100 μM) and incubated for 9 h. Invaded cells were stained and counted under an optical microscope. ** *p* < 0.01, *** *p* < 0.001 compared to control.

To confirm the abilities of EAEC and RA to suppress the migration ability of AGS cells, a wound-healing assay was conducted. Wound closure was detected after 9 h of treatment and compared with that in the untreated control. Treatment with EAEC (10, 25, 50, 100 μg/mL) and RA (10, 25, 50, 100 μM) notably suppressed the migration of AGS cells (Figure 5A). To further confirm the anti-metastatic effects of EAEC and RA on AGS cells, an invasion assay was conducted using a Transwell chamber system coated with extracellular matrix (ECM) gel. Treatment with EAEC (10, 25, 50, 100 μg/mL) and RA (10, 25, 50, 100 μM) markedly inhibited the invasiveness of AGS cells after 9 h of treatment (Figure 5B). These results indicate that EAEC and RA can effectively suppress AGS cell metastasis.

### 2.6. EAEC and RA Modulate the Expression of EMT Regulatory Markers in AGS Cells

**Figure 6:**
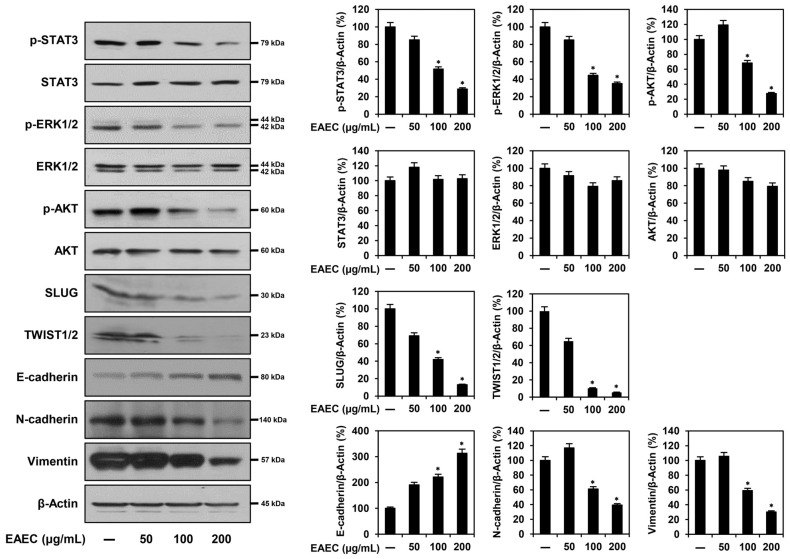

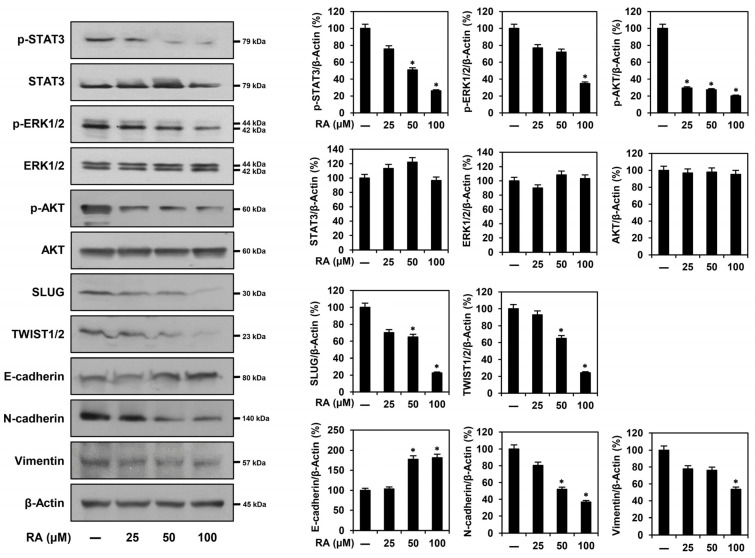
Effects of EAEC and RA on the protein expression of metastasis regulators, evaluated via Western blot analysis. AGS cells were exposed to different concentrations of EAEC (50, 100, 200 μg/mL) and RA (25, 50, 100 μM) and incubated for 24 h. β-Actin served as an internal control. * *p* < 0.05 compared to control.

To assess whether EAEC and RA affect the expression of metastasis-related markers in AGS cells, a Western blot analysis was conducted. As depicted in Figure 6, treatment with EAEC (50, 100, 200 μg/mL) and RA (25, 50, 100 μM) led to a dose-dependent increase in E-cadherin expression and a reduction in N-cadherin and vimentin levels in AGS cells. Treatment with EAEC and RA further lowered the expression levels of upstream markers, including SLUG and TWIST1/2. Moreover, they significantly suppressed the expression of the phosphorylated forms of signaling pathway markers, including STAT3, ERK1/2, and AKT. Overall, EAEC and RA suppressed the metastatic ability of AGS cells by modulating the expression of transcription factors and markers related to EMT through suppression of the STAT3/ERK1/2/AKT signaling cascade.

### 2.7. Inhibitory Effect of RA on AGS Tumor Growth Using a CAM Model

**Figure 7:**
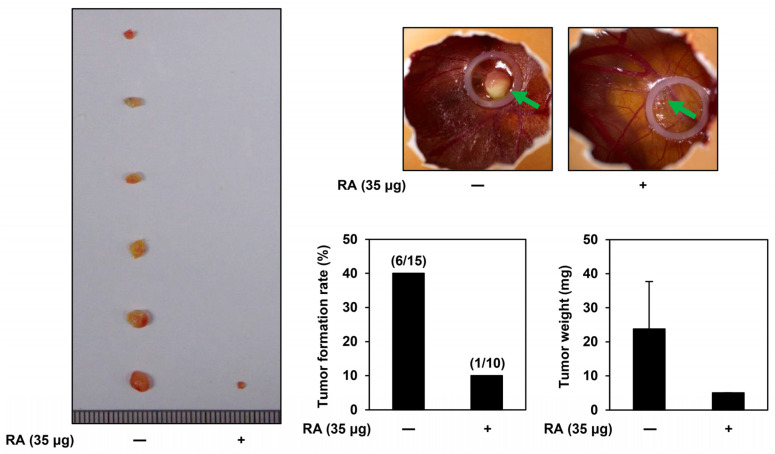
Effects of RA on the in vivo tumor growth in AGS cells, examined using the CAM assay. AGS cells were combined with ECM gel, either with or without RA (35 μg per egg), and injected onto the CAM surface of fertilized chick eggs. After 10 days of incubation, the tumors (green arrows) were excised, and both the tumor formation rate and weight were measured.

A modified chorioallantoic membrane (CAM) assay was used to assess the impact of RA, the main active ingredient in EAEC, on the tumor-forming ability of AGS cells in vivo. A mixture of AGS cells, ECM gel, and RA (35 μg per egg) was injected onto the surface of the CAM and incubated for 10 days. Afterward, the rate of tumor formation and the weights of the formed tumors were analyzed. As depicted in Figure 7, the tumor formation rate was 40% in the control group (6 of 15 eggs) and 10% in the RA treatment group (1 of 10 eggs). Furthermore, the tumor weight was 23.75 mg in the control group and 5 mg in the RA treatment group, indicating that RA treatment significantly suppressed both tumor weight and formation rate. These findings indicate that RA effectively inhibits the tumorigenic ability of AGS cells in vivo.

## 3. Discussion

GC is one of the most prevalent cancers worldwide [1]. Owing to the difficulty of diagnosing GC at its initial stages, most patients are diagnosed in the later stages with metastasis [29]. Therefore, discovering drugs that inhibit both the proliferation and metastasis of GC is a major research priority.

Several anticancer agents have been isolated from natural sources [30]. *E. amoenum* is a well-known medicinal plant, and the phytochemical content and biological activities of its extracts have been revealed [7,11,25]. Additionally, RA has been identified as a major component of *E. amoenum* [12]. Several studies have reported the antioxidant, anti-inflammatory, and anticancer properties of *E. amoenum* and RA. A combination of *Viola odorata, E. amoenum*, and *Physalis alkekengi* extracts is known to effectively improve lower urinary tract symptoms (LUTS) in patients with symptomatic benign prostate hyperplasia (BPH) and is considered a convenient therapeutic option for patients with BPH [31]. In another study, a hydroalcoholic extract of *E. amoenum* was shown to exhibit protective activity against cerulein-induced acute pancreatitis in mice, suggesting its therapeutic potential for the prevention of this inflammatory disease [32]. Furthermore, RA acts as a considerably potent antitumor candidate against colitis-associated colorectal cancer by inhibiting toll-like receptor-4 (TLR4) and myeloid differentiation factor 2 (MD-2) complex-mediated nuclear factor-kappa B (NF-κB) and STAT3 activation [33]. Additionally, RA reduced the malignancy of pancreatic cancer cells through the negative regulation of the Gli1 signaling pathway [34]. A recent study showed that RA affects collagen, matrix metalloproteinases (MMPs), tissue inhibitors of metalloproteinases (TIMPs), glycosylation, and MUC1 in CRL-1739 GC cells, contributing to the suppression of GC metastasis [35]. Furthermore, RA enhances the apoptosis-inducing effects of the anti-MUC1 antibody in AGS GC cells [36]. However, the anticancer and anti-metastatic effects of the ethyl acetate extract of *E. amoenum* (EAEC) and RA on GC, specifically through the inhibition of EMT, have not yet been investigated.

**Figure 8:**
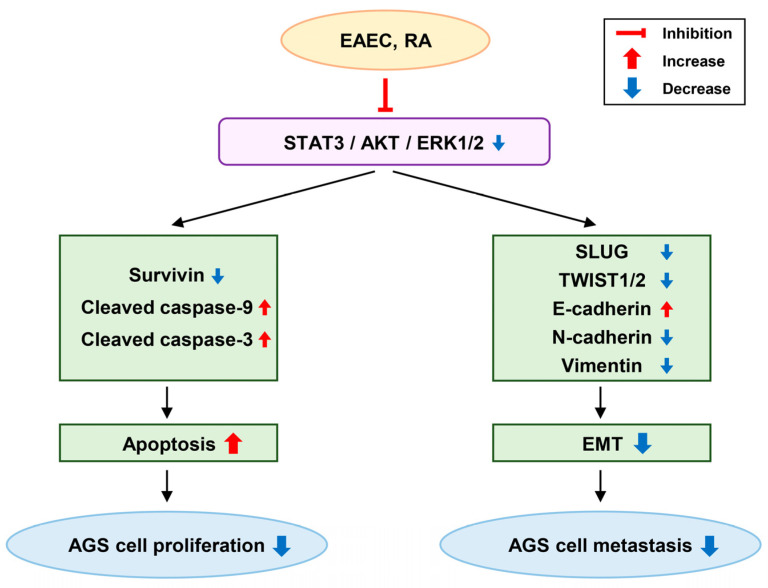
Anticancer mechanism of EAEC and RA in AGS cells.

In this study, the anticancer effects of EAEC were assessed on multiple cancer cell lines, showing the most significant antiproliferative effect in the GC AGS cell line. Accordingly, the anticancer and anti-metastatic activities of EAEC and its active ingredient, RA, along with the molecular mechanisms related to apoptosis and EMT, were further examined in GC cells. The results showed that EAEC and RA suppressed AGS cell proliferation and colony formation. Moreover, both EAEC and RA induced apoptosis by mediating nuclear fragmentation and activating the caspase cascade. Furthermore, EAEC and RA effectively inhibited both cell migration and invasion, which suggested that they suppressed the metastatic ability of AGS cells. The anti-metastatic effect of EAEC and RA against AGS cells is linked to the modulation of major EMT markers and upstream signaling pathways (Figure 8). Furthermore, RA, the primary active component of EAEC, considerably inhibited in vivo tumor-forming ability of AGS cells in the CAM assay.

In GC, the leading causes of death following surgery are cell invasion and metastasis. A key contributor to malignant tumor progression and invasion is the EMT, which is essential for facilitating tumor metastasis [37]. The EMT is a complex restructuring process that enables cancer cells to acquire a metastatic phenotype, characterized by decreased expression of E-cadherin and elevated levels of vimentin and N-cadherin. E-cadherin is crucial for preserving the epithelial phenotype, whereas N-cadherin and vimentin enhance cell motility and invasion [38,39]. The abnormal activation of multiple signal transduction pathways, such as the JAK/STAT3, PI3K/AKT, and MAPK/ERK pathways, promotes cancer cell proliferation and metastasis [40]. STAT3 overexpression enhances cancer cell survival, proliferation, apoptosis resistance, migration, invasion, angiogenesis, immunosuppression, and the self-renewal and differentiation of stem cells by controlling the expression of its downstream target genes. STAT3 also acts as an upstream regulator of the EMT and drives EMT-mediated metastasis in GC cells by influencing the expression of EMT-specific transcription factors [41]. The PI3K/AKT pathway is crucial for enhancing cell survival, as it prevents apoptosis and plays an important role in the EMT, particularly in drug-resistant and metastatic cancer cells [42]. Another study reported that ERK suppression in GC stem cells downregulates the EMT master regulator Snail, along with other mesenchymal markers, leading to decreased migration and invasion [43].

This study demonstrated that EAEC and RA treatment downregulated the STAT3, AKT, and ERK1/2 signaling cascades, which are essential for the growth and metastasis of GC AGS cells. Furthermore, EAEC and RA treatment significantly reduced the expression of the EMT master transcription factors SLUG and TWIST1/2, thereby regulating the expression of essential EMT markers such as vimentin, N-cadherin, and E-cadherin (Figure 8). In summary, EAEC and RA not only inhibited the proliferation of AGS cells by inducing apoptosis but also effectively reduced their metastatic potential by suppressing the EMT. Notably, EAEC and RA demonstrated anti-metastatic activity at lower doses than those required for their antiproliferative effects. Therefore, EAEC and RA hold promise as novel natural product-derived agents for the prevention and treatment of GC, with both anticancer and anti-metastatic activities. However, further research is needed to identify additional active ingredients, besides RA, that may contribute to the inhibitory effects of EAEC on GC cells.

## 4. Materials and Methods

### 4.1. Extraction of E. amoenum

Petals of *E. amoenum* were harvested from Gorgan, Iran, and identified by a botanist (Fereydooni Hossein, PhD, Shahid Chamran University of Ahvaz, Iran). The *E. amoenum* specimen was deposited in the Department of Pharmaceutical Engineering and Biotechnology, Sun Moon University. Ethyl acetate is a highly efficient solvent for extracting a broad range of compounds from plants, owing to its low boiling point, low toxicity, high polarity, selectivity, and capacity to dissolve both hydrophilic and lipophilic substances. To prepare the sample, 10 g of dried *E. amoenum* petals were added to 500 mL ethyl acetate and agitated for 24 h at room temperature. The extract sample was first filtered through gauze, followed by a 0.22 μm syringe filter. The filtrate was reduced in volume using a rotary evaporator and freeze-dried for 4 days. EAEC stock was prepared using dimethyl sulfoxide (DMSO) at a concentration of 200 mg/mL and kept in −20 °C freezer.

### 4.2. Cell Culture

AGS (gastric cancer), HCC827 (lung cancer), Huh7 (liver cancer), U87MG (glioblastoma), and MDA-MB-231 (breast cancer) cell lines were sourced from the Korean Cell Line Bank (Seoul, Republic of Korea). These cells were grown in RPMI-1640, DMEM, or MEM media (HyClone, Marlborough, MA, USA), each supplemented with 10% fetal bovine serum (FBS) (R&D Systems, Minneapolis, MN, USA) and 1% antibiotic/antimycotic solution (Lonza, Walkersville, MD, USA). Cultures were kept at 37 °C in a controlled humidity incubator (Thermo Fisher Scientific, Vantaa, Finland) under a 5% CO_2_ atmosphere.

### 4.3. MTT Assay

AGS, HCC827, Huh7, U87MG, and MDA-MB-231 cells were cultured in 96-well microplates and then exposed to EAEC (0–1000 μg/mL) or RA (0–500 μΜ) (MedChemExpress, South Brunswick, NJ, USA) for 72 h. Cell proliferation was assessed using the MTT colorimetric assay (Sigma-Aldrich, St. Louis, MO, USA) [44]. The IC_50_ values were calculated from the data collected using GraphPad Prism 5 software (La Jolla, CA, USA).

### 4.4. Colony-Forming Assay

To evaluate the impact of EAEC and RA treatment on cell proliferation and colony formation, AGS cells were plated in 6-well plates. After exposing the cells to EAEC (50, 100 μg/mL) or RA (20, 40 μM) for 6 days, the resulting colonies were stained using 0.5% crystal violet (Sigma-Aldrich, St. Louis, MO, USA), and the individual colonies were counted [44].

### 4.5. Apoptosis Analysis

To assess the effect of EAEC and RA on apoptosis, AGS cells were cultured in 60 mm culture dishes and then exposed to EAEC (400, 800 μg/mL) or RA (100, 200 μM) for 24 h. Afterward, the cells were collected, labeled using Muse^®^ Annexin V & Dead Cell reagent (Luminex, Austin, TX, USA), and kept in the dark at room temperature. Apoptosis was evaluated with the Muse^®^ Cell Analyzer using MuseSoft_V1.8.0.3 (Luminex, Austin, TX, USA) [45].

### 4.6. Nuclear Morphology Analysis

AGS cells were placed into 24-well culture plates and exposed to EAEC (400, 800 μg/mL) or RA (100, 200 μM) for 24 h. After treating the cells with 20 μg/mL DAPI (Sigma-Aldrich, St. Louis, MO, USA), nuclear morphology was examined under a fluorescence microscope (Korea Lab Tech, Seong Nam, Republic of Korea) [44].

### 4.7. Intracellular ROS Analysis

AGS cells were plated into 96-well culture plates and exposed to EAEC (400, 800 μg/mL) or RA (100, 200 μM) for 24 h. Following treatment with 20 μM DCFH-DA (Sigma-Aldrich, St. Louis, MO, USA), fluorescence was quantified with a microplate reader (BioTek, Inc., Winooski, VT, USA) [45].

### 4.8. Wound Healing Assay

To create the wound line, Ibidi Culture-Inserts (GmbH, Munich, Germany) were attached to 24-well plates, and AGS cells were added into the inserts. After 24 h of incubation, the inserts were taken out, and the cells were exposed to EAEC (10, 25, 50, 100 μg/mL) or RA (10, 25, 50, 100 μM). After a 9 h incubation, wound closure was observed and captured using an optical microscope (Olympus, Tokyo, Japan). The migration results were quantified as a percentage of the control cell population [46].

### 4.9. Invasion Assay

The lower and upper surfaces of Transwell polycarbonate membrane culture inserts (Corning Costar, Acton, MA, USA) were coated with 10 μL gelatin (1 mg/mL) and 10 μL ECM gel (3 mg/mL) (Sigma-Aldrich, St. Louis, MO, USA), respectively. AGS cells were inoculated into the upper chamber, while EAEC (10, 25, 50, 100 μg/mL) or RA (10, 25, 50, 100 μM) were introduced into the lower chamber containing 600 μL media. After 9 h of incubation, the invaded cells were stained with hematoxylin and eosin (Sigma-Aldrich, St. Louis, MO, USA). The invasion results were quantified as a percentage of the control cell population [46].

### 4.10. HPLC Analysis

To investigate the active ingredient of EAEC, HPLC analysis was carried out using the UltiMate™ 3000 UHPLC (Thermo Fisher Scientific, Waltham, MA, USA). A 40 μL solution of the reference compound RA (100 mM) and EAEC (200 mg/mL) was prepared. HPLC was performed on a Shim-pack GIS C18 column (5 μm, 4.6 mm × 250 mm, Shimadzu Co., Kyoto, Japan) at a UV absorbance of 254 nm and a column temperature of 50 °C. The mobile phase was set at a flow rate of 1 mL/min and consisted of a binary mixture of solvents A (0.1% trifluoroacetic acid (TFA) in water) and B (100% acetonitrile). The elution gradient was as follows: started at 5% B and increased to 90% B from 0 to 28 min, held at 90% B from 28 to 30 min, decreased to 10% B between 30 and 33 min, and maintained at 10% B from 33 to 35 min.

### 4.11. LC-MS Analysis

To confirm the existence of RA (molecular formula: C_18_H_16_O_8_) in EAEC, LC-MS analysis was carried out using an ACQUITY UPLC SYNAPT G2-S QTOF system (Waters, Milford, MA, USA). A 4 μL sample of EAEC (200 mg/mL) and a 1 μL sample of RA (100 mM) were prepared. The binary mobile phase was composed of solvents A (0.1% TFA in water) and B (0.1% TFA in acetonitrile). The column was maintained at 35 °C with a flow rate of 0.3 mL/min. The elution conditions were set as follows: 95% solvent A and 5% solvent B from 0 to 7 min, followed by 0% solvent A and 100% solvent B from 7 to 9.5 min, and then returned to 95% solvent A and 5% solvent B from 9.5 to 12 min. The sample was ionized using the electrospray ionization (ESI) method. After vaporizing at 120 °C and dissolving at 300 °C, the sample was injected into the system under a pressure of 600 L/h and 6.0 bar, where it was analyzed at a capillary voltage of 2.5 kV. Data were analyzed using the UNIFI software (version 1.7, Waters Corporation, Milford, MA, USA). LC-MS raw data are provided in the Supplementary Material. The raw data can be accessed using the program MestReNova (version 14.0.0-23239, Mestrelab Research, Santiago de Compostela, Spain).

### 4.12. Western Blot Analysis

AGS cells were cultured in 100 mm culture dishes and exposed to different concentrations of EAEC or RA for 24 h. The cells were collected and lysed with RIPA buffer (ATTO, Tokyo, Japan). The cell lysates underwent 7.5–15% sodium dodecyl sulfate-polyacrylamide gel electrophoresis (SDS-PAGE) and were transferred onto polyvinylidene difluoride (PVDF) membranes (Amersham Biosciences, NJ, USA). The membranes were blocked for 1 h with a 1–5% skim milk solution in Tris-buffered saline with Tween-20 (TBST), then incubated overnight at 4 °C with specific primary antibodies. After washing with TBST, horseradish peroxidase (HRP)-conjugated secondary antibodies were applied for 1 h at room temperature. Immunolabeling was detected using an ECL kit (DoGeneBio, Seoul, Republic of Korea), and expression levels were quantified by determining the normalized ratio of each target protein to β-actin, with band density analyzed using ImageJ 1.5 software (NIH, Bethesda, MD, USA) [47].

### 4.13. Chick Embryo CAM Assay

To appraise the in vivo anticancer impacts of RA on AGS cells, a CAM assay was conducted [48]. Fertilized chicken eggs were placed in an incubator with controlled humidity at 37 °C. On day 7 of incubation, a small puncture was made in the eggshell under sterile conditions. AGS cells (2 × 10^6^ cells per egg) were then combined with ECM gel (40 μL per egg) and RA (35 μg per egg), and the mixture was subsequently injected into a silicon ring placed on CAM surface. Following 10 days of incubation, the tumors were excised, and both the tumor formation rate along with their weight were recorded.

### 4.14. Statistical Analysis

Results are shown as the mean ± standard deviation (SD) of values from at least three independent experiments. Statistically significant differences between the control and experimental groups were assessed using one-way analysis of variance (ANOVA) with the SPSS 9.0 software (Chicago, IL, USA), followed by Tukey’s post hoc test. A *p*-value of below 0.05 was deemed statistically significant.

## 5. Conclusions

This study aimed to examine the inhibitory effects of EAEC and its primary active compound, RA, on the growth and metastasis of GC AGS cells. EAEC and RA suppressed AGS cell proliferation by inhibiting colony formation and promoting apoptosis. They influenced the expression of apoptosis-related markers, such as survivin, caspase-9, and caspase-3. Additionally, EAEC and RA effectively reduced the migration and invasion abilities of AGS cells. Their anti-metastatic effects are linked to the modulation of EMT-specific markers, including SLUG, TWIST1/2, E-cadherin, N-cadherin, and vimentin. EAEC and RA also disrupted the activation of key upstream signaling pathways, including STAT3, ERK1/2, and AKT, which are crucial in controlling both the proliferation and metastasis of GC cells. Furthermore, RA treatment effectively reduced the in vivo tumor-forming ability of AGS cells in the CAM assay. These findings imply that EAEC and RA may serve as promising agents in anticancer and anti-metastatic therapies for the treatment of GC.

## Data Availability

The data that support the findings of this study are available from the corresponding author upon reasonable request.

## Supplementary Materials

The following supporting information can be downloaded at: https://www.mdpi.com/article/10.3390/ijms252312909/s1.
